# Recent updates and future perspectives about amygdalin as a potential anticancer agent: A review

**DOI:** 10.1002/cam4.2197

**Published:** 2019-05-07

**Authors:** Jiamin Shi, Qianqian Chen, Meng Xu, Qing Xia, Tiansheng Zheng, Junliang Teng, Ming Li, Lihong Fan

**Affiliations:** ^1^ Department of Respiratory Medicine, Shanghai 10th People’s Hospital Tongji University Shanghai China; ^2^ Institute of Energy Metabolism and Health Tongji University School of Medicine Shanghai China; ^3^ Nanjing Medical University Jiangsu China; ^4^ School of information management and engineering Shanghai University of Finance and Economics Shanghai China

**Keywords:** amygdalin, anticancer agents, mechanism, review, traditional Chinese medicine (TCM)

## Abstract

The overall incidence of cancer is increasing in recent years. Despite advances in various comprehensive treatments, the mortality of advanced malignant tumors remains at a high level. Numerous pharmacological studies have confirmed that many Chinese herbal medicines possess remarkable antitumor activities. Amygdalin, mainly existing in bitter almond, is reported to have antitumor properties in addition to the antioxidative, antibacterial, anti‐inflammatory and immunoregulatory activities. This article summarizes the structural characteristics of amygdalin, its antitumor mechanisms, and recent progress and achievement in the research of amygdalin, hoping that it could provide theoretical clues for exploring the clinical value of amygdalin against tumors. Amygdalin is known to have an antitumor effect in solid tumors such as lung cancer, bladder cancer and renal cell carcinoma by affecting cell cycle, inducing apoptosis and cytotoxicity, and regulating immune function. Further research is needed to elucidate the pharmacological mechanisms of amygdalin in terms of the optimal dosage, the feasibility of combined use of amygdalin with other antitumor drugs, and even artificial synthesis of the active components in amygdalin, for the sake of enhancing its antitumor activities and reducing its adverse effects for clinical use.

## 1 INTRODUCTION

Cancer is a major threat to people's health and life worldwide. According to the statistics, approximately 4 292 000 new cases of cancer and 2 814 000 cancer deaths occurred in China in 2015, accounting for about 22% and 27% of the world figures respectively and averaging almost 12 000 new cancer cases and 7500 cancer deaths each day.^1^ Despite the advent of various multidisciplinary tumor treatments including surgery, radiotherapy, chemotherapy and immunotherapy, the mortality rate of patients with advanced malignant tumors remains high.

Natural compounds have been widely accepted and used to treat diseases for centuries. Unremitting efforts made in the field of traditional Chinese medicine (TCM) research in recent years have revealed that many Chinese herbal medicines such as berberine and astragalus have potential antitumor activities by inhibiting tumor progression, regulating immune functions, and attenuating adverse reactions of radiotherapy and chemotherapy.^2^

Amygdalin, one of the main active ingredients of the Chinese raw bitter almond, has been reported to have antioxidative, antibacterial, anti‐inflammatory, and immunoregulatory activities.^3^ As early as 1979, Lea et al^4^ reported the antitumor effect of amygdalin on the Journal of the National Cancer Institute (JNCI). The present review summarizes the structural features, antitumor activities and underlying act mechanisms of amygdalin and recent updates in amygdalin research, hoping that the data provided could provide theoretical clues for exploring the clinical value of amygdalin in terms of its antitumor effect.

## 2 STRUCTURAL CHARACTERISTICS OF AMYGDALIN

**Figure 1:**
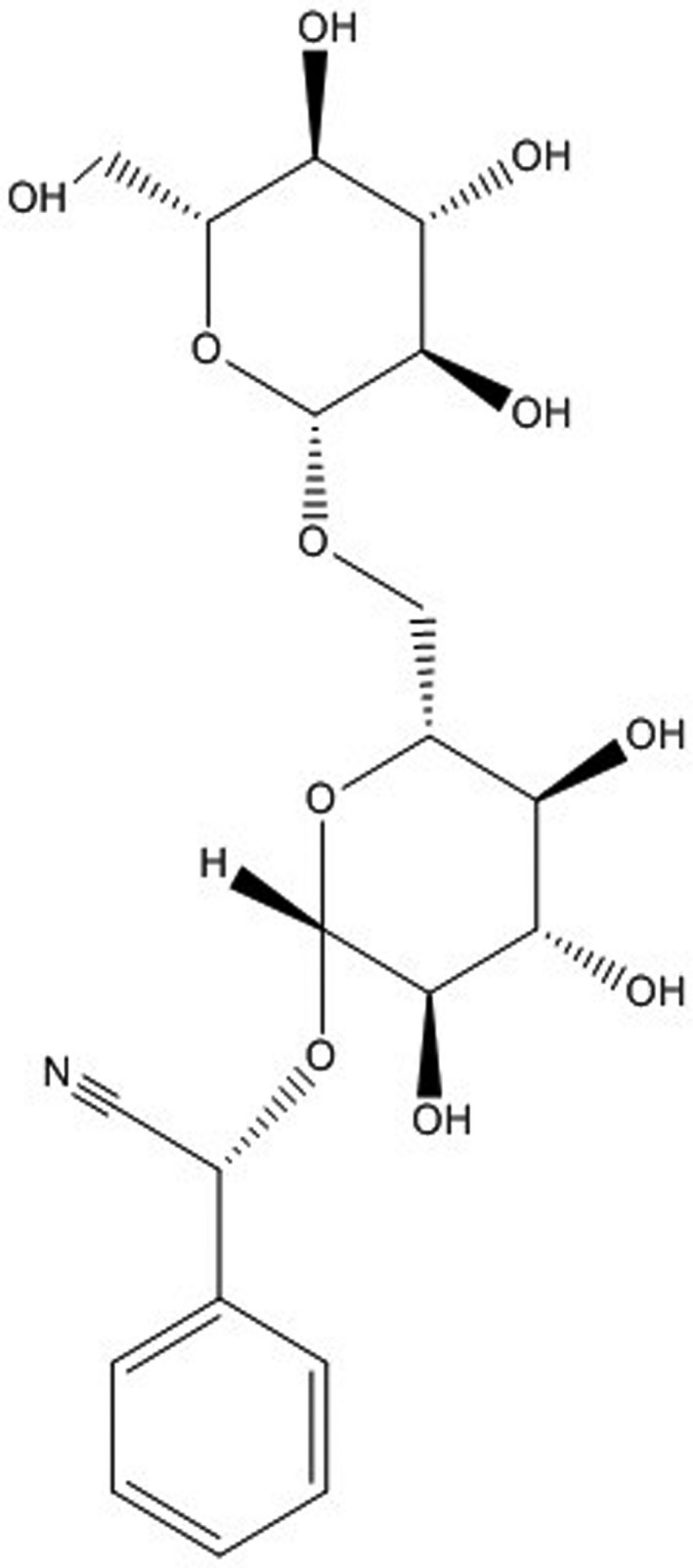
The chemical structure of amygdalin

Amygdalin was first discovered by Schrader et al in 1803,^5^ and in the same year Robiquet et al isolated amygdalin from the bitter almond.^6^ Amygdalin is widely present in the seeds of Rosaceae fruits such as apricots, peaches, and plums,^6^ and mainly in bitter almonds (about 2%‐3%). It is an aromatic aminoglycoside with a molecular formula of C_20_H_27_NO_11_ (Figure 1) and a molecular weight of 457.43, containing one unit of benzaldehyde, one unit of hydrocyanic acid, and two units of glucose.

## 3 THE BASIC ANTITUMOR MECHANISMS OF AMYGDALIN

The antitumor effects of amygdalin are mainly through affecting cell cycle, inducing apoptosis, producing a cytotoxic effect, and regulating the body's immune function (Table 1).

**Table 1:** Specific antitumor mechanisms of amygdalin in different tumors

Types	Cell lines	Dosage of amygdalin	Treatment time	Cellular Effects	Ref.
Lung cancer	H1299	2.5 mg/mL	48 hours	proliferation, invasion, migration	23
	PA	5 mg/mL			
Bladder cancer	UMUC‐3	10 mg/mL	24 hours or 2 weeks	proliferation, adhesion, invasion, migration, cell cycle, cytotoxicity	25, 26, 27
	RT112				
	TCCSUP				
Renal cell carcinoma	Caki‐1	10 mg/mL	24 hours or 2 weeks	proliferation, apoptosis, adhesion, cell cycle	29
	KTC‐26				
	A498				
Prostate cancer	LNCaP	0.1 mg/mL	24 hours	proliferation, apoptosis, cell cycle	7, 10
	DU‐145	1 mg/mL			
	PC3	10 mg/mL			
Cervical cancer	Hela cell	1.25 mg/mL	24 hours	proliferation, apoptosis	9
		2.5 mg/mL			
		5 mg/mL			
		10 mg/mL			
		20 mg/mL			
Colon cancer	SNU‐C4	5 mg/mL	24 hours	proliferation, cell cycle, cytotoxicity	8
Promyelocytic leukemia	HL‐60	1 mg/mL	48 hours	proliferation, apoptosis	30
		2 mg/mL			
		5 mg/mL			
		10 mg/mL			
		20 mg/mL			
Breast Cancer	ER‐positive MCF7	10 mg/mL	24 hours	Cytotoxicity, apoptosis, adhesion	11
	MDA‐MB‐231	20 mg/mL			
	Hs578T	40 mg/mL			

### 3.1 The effect of amygdalin on cell cycle

Makarević et al^7^ exposed prostate cancer (PCa) cell lines LNCaP, DU‐145 and PC3 to different concentrations of amygdalin, and found that cell proliferation was inhibited markedly as represented by a significant decrease in G2/M phase and S phase cells while a significant increase in the number of phase and G0/G1 phase cells by flow cytometry. In addition, the expressions of cell cycle proteins such as CKD1, CKD2, cyclin A, and cyclin B were decreased after amygdalin administration, indicating that amygdalin inhibited cell proliferation by regulating the cell cycle of PCa cells. Similarly, amygdalin exerted its antitumor effect by affecting the cell cycle of human colon cancer. The result of cDNA microarray analysis by Park et al^8^ showed significant differences in gene expression of SNU‐C4 cells after amygdalin treatment at a dose of 5 mg/mL for 24 hours. They found that amygdalin down‐regulated the cell cycle‐related genes: ATP‐binding cassette, exonuclease 1 (EXO1), sub‐family F and topoisomerase (DNA) I (TOP1) in SNU‐C4human colon cancer cells, thereby affecting tumor cell cycle, inhibiting cell proliferation and exerting its antitumor effect. These results demonstrated that amygdalin could prevent malignant proliferation of tumor cells by regulating tumor cell cycle‐related proteins or genes, affecting cell cycle and inhibiting cell proliferation, especially in human PCa and colorectal cancer.

### 3.2 The effect of amygdalin on cell apoptosis

Chen et al^9^ found that amygdalin could promote apoptosis by increasing Caspase‐3 activity in DAPI stained HeLa cells, and the Bcl‐2 was down‐regulated while the Bax was up‐regulated in amygdalin‐treated HeLa cells, suggesting that an intrinsic pathway may be involved in apoptosis. Likewise, amygdalin can also induce PCa cell apoptosis. Chang et al^10^ found that the expression of Bax and the caspase‐3 enzyme activity were increased while the expression of Bcl‐2 was decreased in DU145 and LNCaP cells after amygdalin treatment, leading to PCa cell apoptosis. Lee et al^11^ determined the expression levels of apoptosis‐related proteins in breast cancer cells, which treated with amygdalin at various concentrations, and found that amygdalin increased the expression of pro‐apoptotic protein Bax and decreased that of antiapoptotic Bcl‐2 and pro‐ Caspase‐3. At the same time, PARP cleavage was observed in breast cancer cells treated with amygdalin. These results demonstrate that amygdalin exerts its antitumor effect by regulating apoptosis‐related proteins and inducing apoptosis, especially in cervical and PCa.

### 3.3 The cytotoxic effect of amygdalin

β‐glucosidase is an enzyme that releases glucose by hydrolyzing the glycosidic bond between sugar and aryl groups. The interaction between sugar and aryl groups induces β‐glucosidase to inhibit cytochrome c oxidase which is the terminal enzyme of the mitochondrial respiratory chain, thus terminating the synthesis of adenosine triphosphate and ultimately leading to cell death through catalyzing large amounts of hydrocyanic acid.^12^ Normal cells contain rhodanese to convert hydrocyanic acid to nontoxic hydrocyanic acid, which is absent in tumor cells and can therefore be specifically destroyed by hydrocyanic acid.^13^, ^14^ Li et al^15^ found that the binding between β‐glucosidase and amygdalin could produce a specific antitumor effect. Hydrocyanic acid can act nonspecifically on the cell cycle, thereby killing cancer cells. When the β‐glucosidase is coupled to a tumor‐specific monoclonal antibody, the amygdalin can be converted into an active drug to specifically kill tumor cells. Todorova et al^16^ selected the alkylating agent methyl methanesulfonate (MMS) as a standard mutagen to study the potential antimutagenic effect of amygdalin. Their study found that amygdalin did not have the negative effect on the mutagenic and recombination processes in cells. Meanwhile, lack of toxic effect was observed in normal human cell line, whereas toxicity was determined for the tumor cell lines, indicating the high selectivity of amygdaline toward tumor cells. Furthermore, there are also studies^17^ that found benzaldehyde, a product of amygdalin decomposed by enzymes, can inhibit the activity of pepsin, and is effective for chronic atrophic gastritis which is easily progressing into gastric cancer. These results suggest that a certain concentration of amygdalin can selectively kill tumor cells in the body under the action of β‐glucosidase with insignificant clinical adverse effects, and that amygdalin can generate hydrocyanic acid under the action of human β‐glucosidase and specifically kill tumor cells.

### 3.4 The effect of amygdalin on the body's immune function

The tumor microenvironment^18^ is a site between tumor cells and adjacent normal tissues, where aggregates a large number of immunosuppressive cells such as regulatory T cells, tumor‐associated macrophages, and a large number of inflammatory related factors, such as IL‐6, to promote tumor immunity escape, tumor growth and metastasis. Baroni et al^19^ found that amygdalin can enhance immune function through increasing polyhydroxyalkanoates (PHA)‐induced human peripheral blood T‐lymphocyte proliferation and promoting peripheral blood lymphocytes stimulated by PHA secrete IL‐2 and IFN‐γ to inhibit the secretion of TGF‐β1. The study of Tang et al^20^ indicated that amygdalin treatment can effectively improve the histopathological changes in liver by reducing the levels of malondialdehyde (MDA) and myeloperoxidase (MPO), and alanine transaminase (ALT) and aspartate aminotransferase (AST). Moreover, amygdalin can reduce the secretion of tumor necrosis factor (TNF)‐α, interleukin (IL)‐1β and IL‐6, therefore inhibiting liver inflammation. Zhang et al^21^ found that amygdalin can inhibit inflammation of lung tissue by significantly reducing lipopolysaccharide (LPS)‐induced inflammatory cell infiltration and TNF‐α, IL‐1β, and IL‐6 production and MPO activity in the bronchoalveolar lavage fluid (BALF) through experiments. Therefore, amygdalin can decrease the level of immunosuppressive cells or inflammatory‐related factors to destroy the tumor microenvironment, and inhibit tumor immune escape, tumor growth, and metastasis.

## 4 RECENT UPDATES IN RESEARCH OF THE ANTI‐TUMOR EFFECT OF AMYGDALIN

### 4.1 Lung cancer

Lung cancer is the most common type of cancer in the worldwide.^22^ So we first focused on the role of amygdalin in lung cancer. Qian et al^23^ used amygdalin to treat high metastatic non small‐cell lung cancer (NSCLC) cell lines H1299/M and PA/M, and found that the proliferative, invasive, and migration abilities of H1299/M and PA/M were all inhibited. Amygdalin could also down‐regulate the expression of integrin β1, β4, ILK, FAK, and β‐catenin, factors known to promote cancer cell metastasis, up‐regulate the expression of cadherin E (a factor known to inhibit cancer cell metastasis), and reduce the phosphorylation of AKT and RICTOR, thereby inhibiting the AKT‐mTOR signaling pathway. These results suggest that amygdalin has an antimetastatic effect on NSCLC and exerts its anti‐lung cancer effect by inhibiting AKT‐mTOR signaling pathway.

### 4.2 Bladder cancer

Then we evaluated the antitumor effects of amygdalin in bladder cancer, which ranked the first in the incidence of genitourinary tumors in China.^24^ Makarević et al^25^ detected tumor cell adhesion to vascular endothelial cells or migration of immobilized collagen and tumor cells, and observed the effects of amygdalin on integrin α and β subtypes, ILK (integrin‐linked kinase) and FAK (focal‐adhesion kinase) after treatment of bladder cancer UMUC‐3 and RT112 cells with amygdalin at a dose of 10 mg/mL for 24 hours or 48 hours; they knocked down integrin to evaluate the impact of integrin knockdown on cell migration and adhesion, and found that adhesion and migration of UMUC‐3 and RT112 cells were inhibited after amygdalin treatment, and at the time the expressions of integrin α and β subtypes, ILK and FAK were all decreased.

An in vitro study by Makarević J et al^26^ investigated the effect of amygdalin (1.25‐10 mg/mL) on UMUC‐3, RT112, and TCCSUP bladder cancer cells and found that different concentrations of amygdalin inhibited the proliferation of all the three bladder cancer cell lines to varying degrees, mainly by arresting cell cycle in the G0/G1 phase. Knowing that the optimal effect of amygdalin was observed from CKD2 and Cyclin A, they performed a siRNA knockdown experiment and found that CKD2/Cyclin A expression level was positively correlated with tumor growth, suggesting that tumor growth is probably blocked by amygdalin through reducing CKD2 and Cyclin A.

Amygdalin is also used as a prodrug for antibody‐directed enzyme prodrug therapy (ADEPT) of bladder cancer.^15^, ^27^ Specific activation of amygdalin at the tumor site could kill malignant cells more effectively and reduce toxic side effects associated with conventional chemotherapies. To achieve this purpose, Syrigos et al conjugated β‐glucosidase with a tumor‐associated monoclonal antibody (MAb) (HMFG1) and determined the specificity and cytotoxicity of the conjugate in vitro. It was found that only high concentrations of amygdalin were cytotoxic to HT1376 cells, but exhibited a 36‐fold cytotoxic effect when it was combined with HMFG1‐β‐glucosidase. In addition, they used a MAb‐enzyme conjugate that did not recognize HT1376 cells to demonstrate its specificity and found that amygdalin exerted an anti‐bladder cancer effect by reducing the adhesion and migration of tumor cells, arresting cell cycle progression and binding to HMFG1‐β‐glucosidase.

### 4.3 Renal cell carcinoma

Several studies also showed antitumor effects of amygdalin in renal cell carcinoma (RCC) which ranked the second in genitourinary tumors in China.^28^ Juengel et al^29^ exposed RCC cells A498, Caki‐1and KTC‐26 to amygdalin at a dose of 10 mg/mL for 24 hours or 48 hours and used untreated cells as control. They found that amygdalin reduced the number of cells in G2/M phase (Caki‐1, A498) or S phase (KTC‐26), and increased the number of cells in G0/1 or S phase, and significantly reduced the growth and proliferation of RCC cells. In addition, amygdalin could also markedly reduce cell cycle activator, especially cyclin B, cdk1, E‐ cadherin, and N‐cadherin.

Juengel et al 29 exposed A498, Caki‐1, and KTC‐26 RCC cells to amygdalin and found that amygdalin reduced the chemotactic and invasive activity of tumor cells. FACScan analysis showed that amygdalin down‐regulated integrin α5 and α6, resulting in a marked decrease in tumor cell adhesion to collagen while inhibiting its invasion and metastasis. In summary, amygdalin exerts its anti‐RCC effect by suppressing cell cycle progression, reducing cell cycle‐related proteins, and down‐regulating integrin expression.

### 4.4 Other tumors

**Figure 2:**
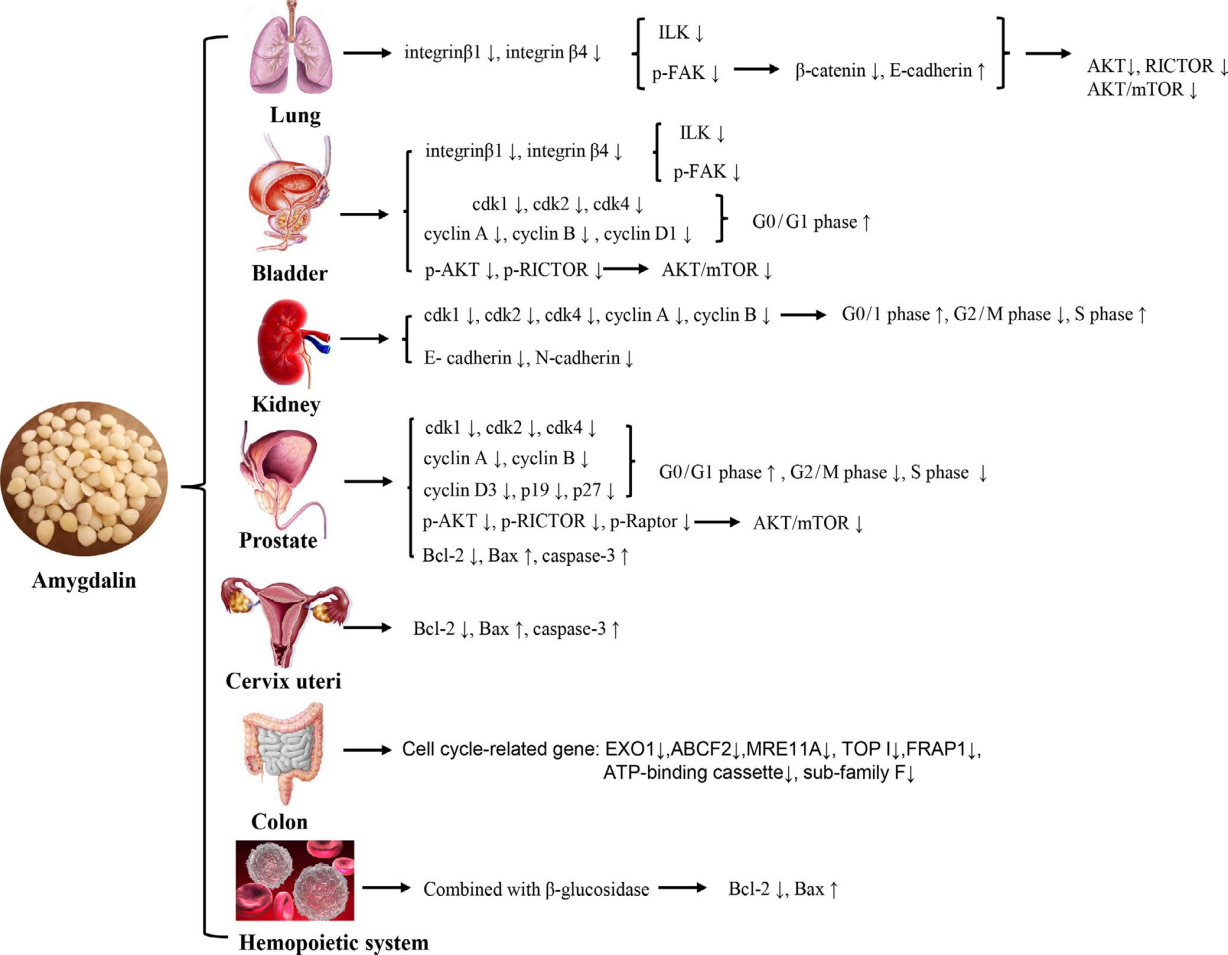
Antitumor effects and mechanisms of Amygdalin

Various studies have shown that amygdalin can inhibit the proliferation, invasion, and metastasis of other malignant tumors in varying degrees (Figure 2).

Several studies reported that amygdalin induced apoptosis and inhibited growth of PCa cells. Chang et al^10^ found that amygdalin up‐regulated Bax, down‐regulated Bcl‐2, and increased the activity of caspase‐3 in DU145 and LNCaP PCa cells. Treatment on PCa DU145 and LNCaP cells with amygdalin increased Bax expression while decreased Bcl‐2 expression. Above all, amygdalin induced apoptosis in human PCa cell lines DU145 and LNCaP by activating caspase‐3, up‐regulating Bax and down‐regulating Bcl‐2. Makarević et al^7^ exposed LNCaP, DU‐145, and PC3 cells to different concentrations of amygdalin for 24 hours or 48 hours and found that the optimal dose of amygdalin to inhibit tumor cell growth was 10 mg/mL, and the number of G2/M phase and S phase cells was decreased while the number of G0/G1 phase cells was increased. Amygdalin could also induce apoptosis of Hela cells in cervical cancer. Chen et al^9^ found that amygdalin significantly inhibited the proliferative activity of cervical cancer HeLa cells. In addition, antiapoptotic protein Bcl‐2 was down‐regulated and pro‐apoptotic Bax was up‐regulated in HeLa cells treated with amygdalin, indicating that apoptosis was involved through the intrinsic pathway.

In human colon cancer cells, amygdalin was also found to reduce the expression of cell cycle‐related genes. Park et al^8^ observed changes in gene expression of SNU‐C4 cells after amygdalin treatment and found that amygdalin down‐regulated cell cycle‐related genes ATP‐binding cassette, exonuclease 1 (EXO1), topoisomerase (DNA) I (TOP1), and sub‐family F.

Amygdalin cytotoxicity can inhibit the proliferation of human promyelocytic leukemia cells. Kwon et al^30^ treated human promyelocytic leukemia HL‐60 cells with amygdalin in combination with β‐glucosidase and found that the cell survival rate was decreased. Also, amygdalin had a cytotoxic effect, thereby inhibiting the proliferation of HL‐60 cells.

## 5 CURRENT STATUS OF CLINICAL RESEARCH ON AMYGDALIN

As described previously, the antitumor effects of amygdalin have been demonstrated in vitro without in vivo experiments. In addition, as described in a previous study,^31^ amygdalin in the doses that they employed produced few clinical side effects. They found that the level of cyanide increased to 2.1 mg/mL in the blood of subjects who received oral amygdalin (0.5 g tid) treatment without showing significant sings of discomfort and all the other parameters were normal, except one patient who developed symptoms of cyanide poisoning due to administration of an excessive dose of amygdalin (1.0 g), in whom the blood level of cyanide reached 3.5 mg/mL. This study showed that amygdalin was safe for patients with advanced cancer.

Shim et al^32^ simulated the digestion of amygdalin by simulating an in vitro digestion model of the human gastrointestinal tract. They found that oral amygdalin was hydrolyzed into prunasin and glucose by digestive enzymes, and then prunasin was degraded to mandelonitrile in the human small intestine which containing β‐glucosidase, and finally taken up as hydroxymandelonitrile with no benzaldehyde or cyanide formed. This result indicated that the toxicity of amygdalin was likely to depend on the microorganisms in the intestine. And then Jaswal et al^33^ found that the amount of hydrogen cyanide (HCN) produced by different intestinal microbes hydrolyzing amygdalin was different. Probiotic consumption, obesity, diet, age, etc all can change the distribution of intestinal microecology, which affects the production of HCN in human body. Therefore, further research is needed to explore the above conditions and to guide the safe oral dose of clinical application of amygdalin.

Several clinical studies were also conducted to evaluate the antitumor effects of amygdalin. The first study about amygdalin in clinical practice was published in the New England Journal of Medicine (NEJM) in 1978.^34^ They assigned 93 patients with different cancers (including lung cancer and lymphoma) into an amygdalin group, a chemotherapy group, and a none‐treatment group, and failed to draw a definite conclusion to support the antitumor activity of amygdalin.

Another study was reported in NEJM in 1982.^35^ It was a descriptive study about the efficacy of amygdalin for 178 cancer patients without a control group. The results suggested that amygdalin had no substantial effect in treating and stabilizing cancer, improving the cancer‐related symptoms, or prolonging life.

These two studies drew the same conclusion that amygdalin was not effective for cancer treatment. However, these two studies had several limitations. They were performed in the last century and were poorly designed. First, they were retrospective studies without providing complete data about the patients involved. The study published in 1982 did not have a control group, which may affect the reliability of the conclusion. Secondly, the sample size of the two studies was not large enough, which may affect power of the conclusion. Finally, for both studies, outcome indicators and the evaluation criteria of the outcomes could be improved to draw a more reasonable conclusion.

To the best of our knowledge, no high‐quality RCTs on amygdalin have been published. To draw a robust conclusion, future multicenter placebo controlled RCTs are required to evaluate the efficacy and safety of amygdalin in cancer treatment.

## 6 CONCLUSION

Previous in vitro studies demonstrated that amygdalin exerted its antitumor effect by affecting cell cycle, inducing apoptosis and cytotoxicity, and regulating immune function. However, clinical trials found that amygdalin metabolites may convert to hydrocyanic acid, and accumulation of hydrocyanic acid over time may produce an adverse toxic effect on the human body.

## 7 SUMMARY AND OUTLOOK

This study is unique in several aspects. First, we have included all relevant published studies in the literature. Second, we have comprehensively described the antitumor mechanisms of amygdalin by citing experiments both in vivo and in vitro. Finally, we summarize the conclusions of these studies in an objective and comprehensive way. Proper figures and tables are also provided to demonstrate the specific antitumor mechanisms of amygdalin in different tumors.

However, this review also has some limitations. First, the experimental and clinical research on amygdalin is still in the primary stage, and no high‐quality articles have been published. Second, the retrieved studies on amygdalin were performed in different types of tumors, and few further studies were performed to evaluate specific molecular mechanisms. Future studies should focus on specific antitumor mechanisms of amygdalin in different tumor types.

BATMAN‐TCM, an online bioinformatics analysis tool for TCM molecular mechanisms. Adenosine A2A (ADOA2A), adenosine A2B (ADOA2B), adenosine receptor A3 (ADOA3) and adenosine receptor A1 (ADOA1) were detected by this tool (http://bionet.ncpsb.org/batman-tcm/index.php/Home/Index/target/jobId/batman-I2018-03-06-21011-1520311395/cutoff/20/pVal/0.05).

In addition, there are 56 membrane receptors of amygdalin, including ADOA2A, ADOA3, and ADOA1, as well as their top biological activity by ChEMBL (Chemical Database) (https://www.ebi.ac.uk/chembl/target/results/keyword).

Adenosine receptors have also been the research hotspot in recent years. They have multiple therapeutic effects, especially in the treatment of cancer. Even targeted therapeutic drugs have been synthesized and are at the experimental stage.

Few studies on the target of amygdalin have been reported, and therefore more studies are required to elucidate the pharmacological mechanisms of amygdalin in terms of the optimal dose, the feasibility of combined use of amygdalin with other anti‐tumor drugs, and even artificial synthesis of the active components in amygdalin, for the sake of enhancing its antitumor effect and reducing its adverse effects for clinical use.
